# Association Between Maxillary Labial Frenum Attachment Types and Early Childhood Caries: A Cross-Sectional Study

**DOI:** 10.3290/j.ohpd.c_2277

**Published:** 2025-09-26

**Authors:** Nurettin Yusuf Yilgör, Sera Şimşek Derelioğlu, Pınar Eser Tuna, Fatih Şengül, Nazlı Nur Aslan İnce

**Affiliations:** a Nurettin Yusuf Yilgör Private Dentist, Denizli, Turkey. Idea conception, data collection.; b Sera Şimşek Derelioğlu Associate Professor. Department of Pediatric Dentistry, Faculty of Dentistry, Atatürk University, Erzurum, Turkey. Idea conception, wrote the manuscript.; c Pınar Eser Tuna PhD student, Department of Periodontology, Faculty of Dentistry, Atatürk University, Erzurum, Turkey. Wrote the manuscript.; d Fatih Şengül Assistant Professor. Department of Pediatric Dentistry, Faculty of Dentistry, Atatürk University, Erzurum, Turkey. Data analysis.; e Nazlı Nur Aslan İnce PhD student, Department of Pediatric Dentistry, Faculty of Dentistry, Atatürk University, Erzurum, Turkey. Data analysis.

**Keywords:** early childhood caries, maxillary labial frenum, early frenectomy

## Abstract

**Purpose**: This study aimed to evaluate the distribution of maxillary labial frenum (MLF) attachment types and their potential association with the development of early childhood caries (ECC) in children aged 2 to 5 years who presented to our clinic for routine check-ups or treatment.

**Materials and Methods:**

A total of 92 children aged 24–71 months (53 girls and 39 boys) were included in the study. Clinical oral examinations were performed, and MLF attachment types were recorded using Placek’s classification. Standardised intraoral photographs were taken to document the anterior maxillary region and frenum attachments. Children were categorised into ECC and caries-free groups based on clinical findings. Statistical analyses were conducted using Statistical Package for the Social Sciences (SPSS) v26.

**Results:**

The mean age of the participants was 52.7 ± 11.7 months. In the ECC group, the mean dmft score was 9.8 ± 4.3. Although no statistically significant relationship was found between overall frenum attachment types and caries formation in the anterior maxillary region, papillary and mucosal attachment types were more common in children with anterior caries, while the gingival type was more prevalent in the caries-free group (P < 0.05).

**Conclusion:**

A statistically significant association was observed between certain MLF attachment types and anterior caries; however, the presence of caries in mucosal-type attachments – typically considered low-risk – suggests that other aetiological factors play a more prominent role in ECC development. Therefore, clinicians should focus on comprehensive caries risk assessment before considering surgical interventions such as frenectomy.

Early childhood caries (ECC), the most prevalent chronic disease of childhood, continues to develop at an increasing rate despite various efforts in prevention and treatment. Researchers have identified several risk factors for ECC, including maternal active caries, frequent consumption of sugary foods and snacks, intake of sweetened beverages before or during sleep, passive smoking, special healthcare needs, xerostomia, and inadequate fluoride exposure.^1,2, 3, 6, 7, 8, 9,10,13,22^

In addition to clinical factors such as visible plaque accumulation and enamel hypoplasia,^4^ anatomical structures, like the maxillary labial frenum (MLF), have recently gained attention as potential contributors to ECC.^11,13^ Abnormal frenum attachment may hinder effective oral hygiene in the anterior region, facilitating plaque retention and increasing caries susceptibility. Moreover, social determinants such as low socioeconomic status and limited parental education and health literacy remain significant risk factors for ECC.^5,14^

Although a few studies have examined the relationship between MLF attachment types and dental caries,^16^ there is still a lack of research focusing specifically on the association between MLF morphology and ECC, especially studies that take multiple confounding factors into account.^15,23^

Therefore, the aim of the present study was to investigate whether the distribution and attachment types of the MLF were associated with the development of ECC in children aged 2 to 5 years who presented to our clinic for treatment or routine check-ups.

The null hypothesis was that the types and morphology of the maxillary labial frenum are not associated with early childhood caries.

## MATERIALS AND METHODS

### Study Consent

This descriptive cross-sectional study was designed to assess MLF during oral examinations of children aged 2–5 years who applied to Atatürk University, Faculty of Dentistry, Department of Pedodontics for dental treatments or check-ups. And by using the data obtained, we planned to search whether the different attachment types of MLF had any impact on the formation of ECC or not.

### Ethical Committee Approval and Official Consents

This study was conducted by the Department of Pedodontics, Atatürk University, in accordance with the provisions of the Ministry of Health Clinical Research Regulations and with the Faculty of Medicine Research Ethics Committee‘s written approval (session No.07/11/2019 resolution #07/36).

### Participant Recruitment

This study was designed as a cross-sectional observational study. As no intervention or comparison between treatment groups was involved, an *a priori* power analysis was not performed. The sample size was based on the availability of eligible participants during the study period.

Children with no clinically detectable carious lesions on any tooth surface, based on visual-tactile examination using a mirror and probe, were included in the caries-free group. No radiographs were taken. All children who met the inclusion criteria and attended the clinic during the study period were invited to participate (convenience sampling).

#### Inclusion criteria

2–5-year-old children with or without ECC and accompanied by their legal guardians

#### Exclusion criteria

Children whose parents are unwilling to participate.
Children with any systemic disorder that interferes with oral hygiene.
Children with any mental or physical disability.
Children with cleft lip and palate, congenital deformities, orofacial syndromes with a history of surgical intervention or trauma in the maxillary labial region, with past medication use, which were known to have adverse effects on the gingiva.

### Data Collection

Study data were gathered through the oral examination forms, which represented the patients’ current oral health status, consisting of questions used for determining caries prevalence and some risk factors and also through the intraoral photographs taken during dental examinations.

#### Man power

Observer training and calibration are crucial for obtaining consistent examination data and conducting accurate and reliable epidemiological research.^15^ In our study, all the examinations were performed by a single examiner who was a resident in pedodontics. Prior to the study, this examiner was trained by another senior researcher registered in paediatric dentistry.

Intra and inter-examiner reliability tests were performed in order to provide data consistency and reproducibility. The same researcher evaluated the intra-examiner reliability by re-reviewing the MLF images and dmft scores of 20 patients, two weeks apart. Another researcher tested the inter-examiner reliability independently by evaluating the dmft scores and MLF images of the same 20 patients.

#### Data collection method

Data obtained from the child patients and oral findings were recorded on the examination forms. Collected data were analysed with IBM® Statistical Package for the Social Sciences (SPSS® v20).

### Clinical Investigation

This section includes the types of anatomical attachment of MLF and dmft scores, assessing the oral health status.

#### Determining the attachment types of MLF

In a dental chair unit (DCU), while mothers held the children in their laps, two dentists retracted patients’ labials, and the third one took intraoral photographs. Then, the images were recorded, and frenum attachment types were assessed after all the study data were gathered. Firstly, one examiner completed the image evaluations and noted the types of frenum of all children.

**Fig 1a to d:**
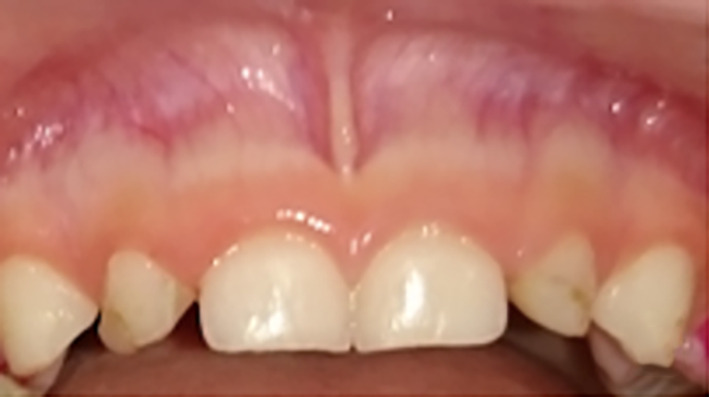

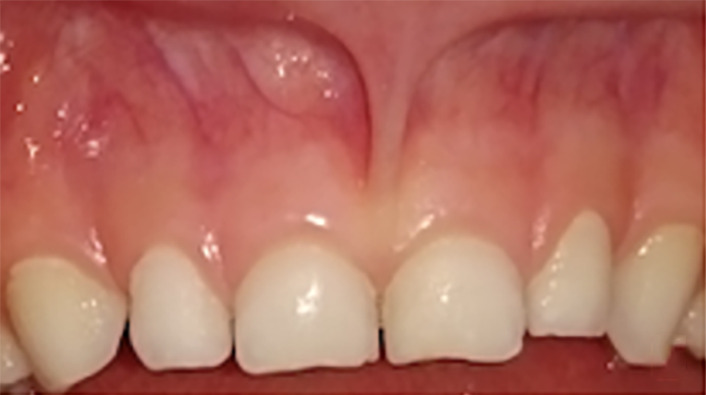

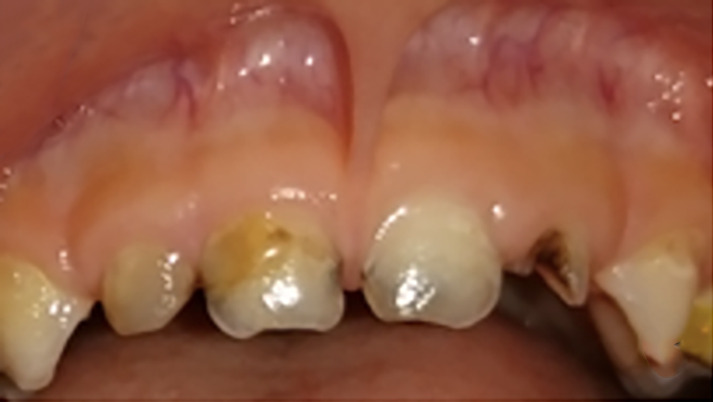

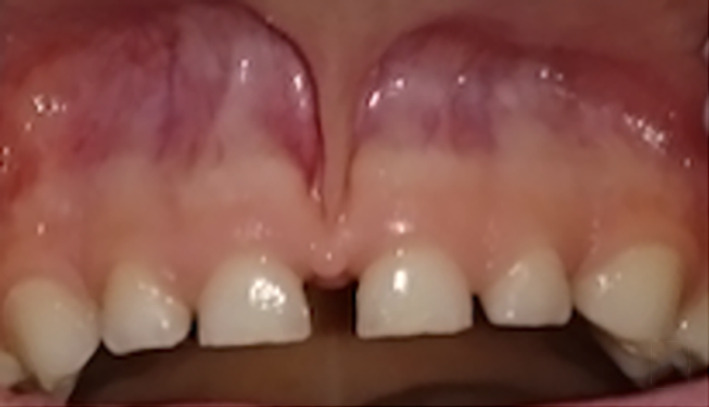
Placek’s classification of maxillary labial frenum attachment. (a) Mucosal attachment; (b) gingival attachment; (c) papillary attachment; (d) papillary penetrating attachment. (Images were taken from the participating patients’ intraoral photographic database).

Subsequently, the second assessment evaluated the intraoral photographs, and the patients’ frenum types and morphologies were also recorded. Later, both examiners cross-checked their assessments and reached a consensus by jointly re-reviewing the differently assessed images. Placek’s classification of MLF connection types^18^ is shown in Figure 1.

#### Caries detection method

Dental examinations were conducted in a DCU under proper lighting using a World Health Organization (WHO) community periodontal index (CPI) probe (WHO 973/80 – Martin, Solingen, Germany) and a mouth mirror. Caries detection was performed based on WHO diagnostic criteria (visual-tactile method) without the use of radiographs. The findings were used for dmft scoring.

### Statistical Analysis

Study data were analysed with SPSS® v20, categorical variables were expressed as counts and percentages and numerical variables were presented as means ± standard deviation (SD). After the Chi-square analysis was found to be statistically significant, the Post Hoc test was used to uncover which cells larger than 2×2 were different from their expected values. Kruskal–Wallis and Mann–Whitney U tests were used for inter-group comparisons of non-normally distributed numerical variables. Intra- and inter-reliability were evaluated with Kappa statistics. P <0.05 was accepted as statistically significant.

## RESULTS

This study involved a total of 92 child patients with a mean age of 52.7 ± 11.7 months (minimum 24 and maximum 71 months), of whom 53 were girls (57.6%) and 39 boys (42.4%). The mean age of the girls and boys was 52 ± 12 and 54 ± 11.4 months, respectively.

The mean maternal education level of the children was determined as 9.4 years (approximately middle school graduate), whereas the mean educational level of the children’s fathers was observed to be 11.8 years (approximately high school graduate), which was higher than that of the mothers (P <0.001).

When we assessed parental social status and examined the percentage of maternal employment, we determined that 20.7% of the mothers (19 mothers) were working and 79.3 of them (73 mothers) were non-working. 85.9% of the children (79 toddlers) were parented by their mothers, 7.6% of them (7 toddlers) were fostered by their grandmothers, and 6.5% (6 toddlers) by babysitters. In terms of parental income, we found that 56.5% of the families’ (52 families) revenues were below the national minimum wage, and 43.5% of them (40 families) earned higher than the minimum wage.

**Fig 2:**
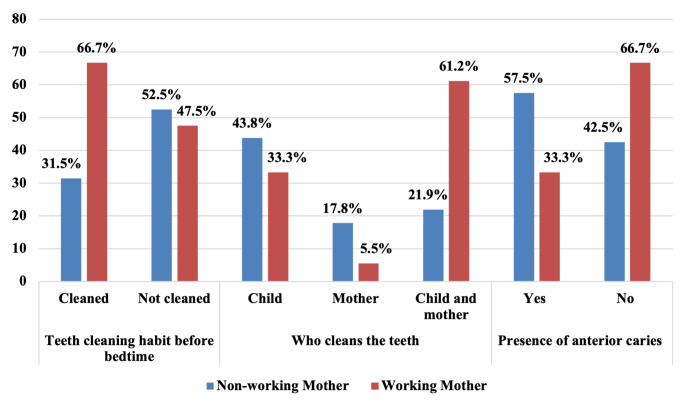
Correspondence between the maternal employment and some caries-related parameters.

Influence of maternal employment on the children’s maxillary anterior caries, their tooth brushing habits before bedtime and by whom the teeth were brushed are given in Figure 2. The percentage of maxillary anterior caries in working mothers was found to be significantly lower (31.6%) than that of non-working mothers (57.5%) (X^2^ (1, N = 92) = 4.070, P = 0.04). When tooth brushing habits before bedtime were evaluated, we observed that 34.3% of the children of the employed mothers brushed their teeth before sleep, while 65.7% of the unemployed mothers’ children had a tooth brushing routine before bedtime. A higher rate of tooth brushing routine was seen in the children of the working mothers, which was found to be statistically significant (X^2^ (1, N = 92) = 4.725, P = 0.030). When we researched who brushed the children‘s teeth, we found a higher parental-supervised toothbrushing rate in the children of the working mothers (61.1%). In the children of the non-working moms, a higher rate of teeth brushing performed by the children themselves (43.8%) than by the mothers only (17.8%) and supervised brushing (21.9%) was observed to be a statistically significant difference (X^2^ (2, N = 92) = 7.952, P = 0.019).

65.2% of the participating patients (60 children) breast-fed until 2 years of age, and 34.8% of them (32 children) stopped breastfeeding before they had reached the age of 1. 35.9% of the participants (33 children) have used a pacifier, and 15.1% of them (5 children) used a sweetened pacifier. 46.7% of the participants (43 children) were bottle-fed, whereas 53.3 % of them (49 children) were not.

85.9% of the patients (79 children) had a toothbrushing habit, with a rate of 44.3% brushing before bed routine (35 children). Fluoride was applied at least once to only 13% of the participants (12 children), and 87% of them (80 children) did not receive fluoride varnish application (Table 1).

**Table 1:** Children’s status of nutrition, dental hygiene behaviours and fluoride application

	n (%)		n (%)
Duration of breastfeeding	Pacifier usage?
≤1 year of age	32 (34.8%)	Yes	33 (35.9%)
≤2 years of age	60 (65.2%)	No	59 (64.1%)
**Sweetened pacifier?**	**Duration of pacifier usage**
Yes	5 (15.1%)	Night-time only	14 (42.4%)
No	28 (84.9%)	All day	19 (57.6%)
**Bottlefeeding**	**Duration of bottlefeeding**
Yes	43 (46.7%)	Night-time only	16 (37.2%)
No	49 (53.3%)	All day	27 (62.8%)
**Contents of the feeding bottles**	**Reasons for bottlefeeding**
Foods containing sweeteners	12 (27.9%)	Particular challenging feeding issues	10 (23.2%)
Infant formula	6 (13.9%)	Supplementary feeding	23 (53.5%)
Plain milk	14 (32.6%)	As a routine dietary behaviour	10 (23.3%)
Infant formula and milk	11 (25.6%)		
**Toothbrushing?**	**Who brushes the teeth?**
Yes	79(85.9%)	Child	38 (48.1%)
No	13 (14.1%)	Parent	14 (17.7%)
		Child+parent	27 (34.2%)
**Toothbrushing tools**	**Brushing before bed?**
Only toothbrush	7 (8.9%)	Yes	35 (44.3%)
Toothbrush + toothpaste	72 (91.1%)	No	44 (55.7%)
**Daily toothbrushing frequency?**	**Fluoride application?**
Occasionally or none	34 (43.1%)	Yes	12 (13%)
More than once a day	45 (56.9%)	No	80 (87%)
n: Number

Almost a perfect agreement between inter-and intra-rater reliability was observed in the evaluation of dmft scores and MLF (κ_dmft-inter_ = 0.96, κ_MLF-inter_ = 0.89 and κd_mft-intra_ = 0.92, κ_MLF-intra_ = 0.87).

Table 2 illustrates the relationship between eruption time and dmft/dmfs scores of maxillary anterior caries status. Mean dmft and dmfs index scores for children’s maxillary anterior teeth (MAT) were calculated as 6.8 ± 4.8 (min 0, max 20) and 13 ± 14.1 (min 0, max 88), respectively. dmft (8.2 ± 5.5) and dmfs index scores (18.3 ± 18.8) of the children whose teeth erupted before 7 months were found to be higher than those of the ones with relatively earlier eruption time (p_dmft_ = 0.032, p_dmfs_ = 0.007).

**Table 2:** Correlation between eruption time and caries status

	n	dmft M ± SD	dmfs M ± SD	P value
**Tooth eruption time**				
**Before 7 months**	33	8.2 ± 5.5	18.3 ± 18.8	p_dmft_ = 0.032 p_dmfs_ = 0.007
**After 7 months**	59	6 ± 4.3	10.1 ± 9.7
**Total**	92	6.8 ± 4.8	13 ± 14.1	
**Presence of anterior caries**				
**Yes**	48	9.8 ± 4.3	20.1 ± 16	p_dmft_ <0.001 p_dmfs_ <0.001
**No**	44	3.5 ± 2.8	5.3 ± 5.2
n: Number, M: Mean, SD: Standard deviation.

Lower mean dmft (3.5 ± 2.8) and dmfs (5.3 ± 5.2) index scores of the child patients having no caries in their MAT than the children with MAT caries were statistically significant (p_dmft_ <0.001, p_dmfs_ <0.001) (Table 2).

Lower mean eruption time in the patients with no anterior caries (9.1 ± 2.7 months) than the patients experiencing anterior caries (7.7 ± 2.4 months) was observed to be statistically significant (P = 0.008). Moreover, a higher rate of MAT caries (67%, n = 22) in the children whose teeth erupted earlier than 7 months, and increased rates of non-existed carious anterior teeth (56%, n = 33) in the child patients experienced tooth eruption after the 7th month (X^2^ (1, N = 92) = 4.331, P = 0.037).

The relationship between the number of carious teeth (d) detected in clinical examinations and sweetened pacifiers, maternal employment, and caregivers is shown in Table 3. Among the pacifier-using children, the mean number of carious teeth (9.2, 6.8) in the children using sweetened pacifiers was higher than in those not using sweetened pacifiers (4.8, 3.7) (P = 0.039). Mean d value of the employed mothers’ children (2.4, 2.9) was found to be significantly lower than the children of unemployed mothers (6.6 ± 4.6) (P <0.001). When caregivers were assessed, the mean d value of the children living under maternal care (6.3 ± 4.7) was higher than that of the children fostered by grandmothers (2.6 ± 3.6) and babysitters (2.8 ± 2.6) (P = 0.033).

**Table 3:** Distribution of the mean number of carious teeth(d) by sweetened pacifiers, maternal employment and caregivers

	n	d (M ± SD)	P value
**Sweetened pacifier?**			
Yes	5	9.2 ± 6.8	P = 0.039
No	28	4.8 ± 3.7	
Total	33	5.4 ± 4.5	
**Working mother?**			
Yes	19	2.4 ± 2.9	P <0.001
No	73	6.6 ± 4.6	
Total	92	5.8 ± 4.6	
**Caregiver?**			
Mother	79	6.3 ± 4.7	P =0.033
Grandmother and others	7	2.6 ± 3.6	
Baby-sitter and others	6	2.8 ± 2.6	
Total	92	5.8 ± 4.6	
n: Number, M: Mean, SD: Standard deviation

Table 4 represents the attachment types of frenum. Predominantly, gingival-type frenal attachment was observed in 68.5% of the participants. Higher frequency rates of mucosal and papillary frenum types seen in the children with maxillary anterior caries and gingival frenum type observed in the children with no anterior caries were found to be statistically significant X^2^ (2, N = 92) = 6.982, P = 0.03).

**Table 4:** Distribution of maxillary anterior caries by frenal attachment types

Attachment type	n (%)	Maxillary anterior caries	P value
No n (%)	Yes n (%)
Mucosal	21 (22.8%)	6 (13.6%)	15 (31.3%)	P = 0.03
Gingival	63 (68.5%)	36 (81.8%)	27 (56.2%)
Papillary and papillary penetrating	8 (8.7%)	2 (4.6%)	6 (12.5%)
Total	92 (100%)	44 (100%)	48 (100%)
n: Number

## DISCUSSION

This study investigated whether the attachment types of MLF were associated with ECC. The findings revealed a statistically significant difference in the distribution of frenum attachment types between children with and without maxillary anterior caries. Specifically, papillary, papillary penetrating, and mucosal attachment types were more common among children with anterior caries. In contrast, the gingival type was predominantly found in caries-free individuals.

Interestingly, although papillary and papillary penetrating types are anatomically closer to the gingival margin and are hypothesised to hinder oral hygiene, the mucosal type – considered the lowest risk in terms of plaque retention – was also associated with a high rate of anterior caries in our study. This unexpected outcome suggests that frenum attachment alone may not be a sufficient aetiologic factor for ECC and that other predisposing factors likely play a more prominent role.

Based on these findings, the null hypothesis – that MLF types are not associated with ECC – was rejected.

Our results partially align with Kotlow’s theory that thick, fibrotic, and papillary-type frenula can predispose anterior teeth to decay by creating a niche for milk retention in breastfeeding infants.^15,16^ However, our study also showed a high frequency of caries in children with mucosal-type frenum, challenging the idea that only more invasive types (eg, papillary penetrating) pose a significant risk. Similarly, Kılınç et al^13^ reported no significant relationship between frenum type and anterior caries, which supports the notion that other contributing factors may be more critical in ECC pathogenesis.

In contrast, Taran et al^21^ reported a statistically significant association between papillary-type MLF and the presence of anterior caries in preschool children, consistent with part of our findings. However, their study did not discuss mucosal-type attachments, which were also associated with high caries prevalence in our sample. Kaplan and Taraç^12^ also emphasised the distribution of MLF types in children and noted a potential clinical impact on oral health, though they did not directly evaluate caries outcomes. Thus, our study adds to the literature by specifically examining ECC development in relation to different frenum attachment types, including the less commonly analysed mucosal form.

In addition to anatomical variations, the present study also explored the influence of socioeconomic and behavioural factors on ECC development. Although variables such as maternal education level, household income, and maternal employment status were not significantly associated with ECC in our sample, some trends were observed. For instance, children of mothers with higher education levels tended to have lower mean dmft scores, and children from families earning above the minimum wage showed a slightly reduced prevalence of caries.

These findings are consistent with previous research indicating that higher socioeconomic status and parental education are associated with better oral health outcomes in children.^9,19,20,23^ Similarly, supervised toothbrushing – more frequently observed in children of working mothers—was associated with improved oral hygiene and lower caries incidence. However, due to the lack of statistical significance, these associations should be interpreted cautiously and warrant further investigation with larger sample sizes.

In our study, it was observed that children of employed mothers had lower mean dmft scores and were more likely to receive parental assistance with toothbrushing, particularly before bedtime. This finding may reflect greater health literacy and oral health awareness among working mothers. Parental-supervised brushing, as reported in previous studies,^6^ plays a key role not only in improving brushing effectiveness but also in establishing a long-term toothbrushing habit in children.

In the present study, we also evaluated the relationship between tooth eruption timing and ECC. Children whose maxillary anterior teeth erupted earlier than seven months had higher mean dmft and dmfs scores compared to those with later eruption. This may be attributed to increased exposure duration to cariogenic factors in early-erupting teeth. Conversely, lower caries rates in children with delayed eruption were expected, as these teeth had a shorter time window for plaque accumulation and dietary exposure.

In the literature, there are several studies representing the impact of night-time bottlefeeding and pacifier usage on ECC development.^2,21^ Also, in the present study, an elevated rate of caries was seen in the children who used sweetened pacifiers, which was found to be statistically significant.

While some authors, including Kotlow,^16^ advocate early frenectomy in infants with high-risk frenum types, others such as Nathan^17^ argue that surgical intervention should be delayed until natural anatomical changes occur around the age of 9–11 years. Nathan emphasises that spontaneous repositioning of the frenum and physiological closure of midline diastema due to the eruption of permanent canines may reduce or eliminate the need for frenectomy.

Our study also evaluated other contributing factors, including socioeconomic status, maternal education, oral hygiene practices, and feeding behaviours. Although trends were observed, such as lower dmft scores in children of working or more educated mothers, these were not statistically significant. Nonetheless, supervised brushing and reduced pacifier use, especially with sweetened substances, were associated with lower caries rates.

The main limitation of this study was the relatively small sample size, which restricts the generalizability of the findings. Additionally, the lack of an *a priori* power analysis should be acknowledged as a methodological limitation. Larger, multicentre studies are needed to validate the association between specific frenum types and ECC development.

## CONCLUSION

Although abnormal frenum attachments may play a role in plaque retention and caries susceptibility, they should not be considered in isolation when evaluating ECC risk. Before considering surgical procedures such as frenectomy – which may be traumatic for infants and young children – other modifiable risk factors should be carefully assessed and managed. Early dental visits and regular follow-up care can facilitate individualised preventive strategies, including fluoride application, oral hygiene education, and dietary counselling to support optimal oral health in children.

### Acknowledgements

The data sets generated and analysed during the current study are available from the corresponding author on reasonable request. All procedures performed in studies involving human participants were in accordance with the ethical standards of the institutional and/or national research committee and with the 1964 Helsinki Declaration and its later amendments or comparable ethical standards. The study was approved by the Bioethics Committee of the Medical University of Atatürk (session No.07/11/2019 resolution #07/36).

Informed consent was obtained from the parents of all children included in the study.

Written informed consent for publication of intraoral photographs was obtained from the parents or legal guardians of all child participants. The images included in the manuscript contain only teeth and the labial frenum, and do not allow personal identification.

### Disclosure

The authors declare no competing interests.

## References

[ref1] Aliakbari E, Gray-Burrows KA, Vinall-Collier KA, Edwebi S, Marshman Z, McEachan RRC, Day PF (2021). Home-based toothbrushing interventions for parents of young children to reduce dental caries: a systematic review. Int J Paediatr Dent.

[ref2] American Academy of Pediatrics (2014). Section on Oral Health. Maintaining and improving the oral health of young children. Pediatrics.

[ref3] Bissar A, Schiller P, Wolff A, Niekusch U, Schulte AG (2014). Factors contributing to severe early childhood caries in south-west Germany. Clin Oral Invest.

[ref4] Caufield PW, Li Y, Bromage TG (2012). Hypoplasia-associated severe early childhood caries: a proposed definition. J Dent Res.

[ref5] Congiu G, Campus G, Lugliè PF (2014). Early childhood caries (ECC) prevalence and background factors: a review. Oral Health Prev Dent.

[ref6] Davies GN (1998). Early childhood caries – a synopsis. Community Dent Oral Epidemiol.

[ref7] Douglass JM, Li Y, Tinanoff N (2008). Association of mutans streptococci between caregivers and their children. Pediatr Dent.

[ref8] Drummond B, Milne T, Cullinan M, Meldrum A, Coates D (2017). Effects of environmental tobacco smoke on the oral health of preschool children. Eur Arch Paediatr Dent.

[ref9] Dye BA, Shenkin JD, Ogden CL, Marshall TA, Levy SM, Kanellis MJ (2004). The relationship between healthful eating practices and dental caries in children aged 2–5 years in the United States 1998–1994. J Am Dent Assoc.

[ref10] Fontana M (2015). The clinical, environmental, and behavioral factors that foster early childhood caries: evidence for caries risk assessment. Pediatr Dent.

[ref12] Kaplan TT, Taraç MG (2025). Maxillary labial frenum types in children and effects on the oral cavity: a cross-sectional study. ADO Klinik Bilimler Dergisi.

[ref13] Kılınç G, Bulut G, Ertuğrul F, Ersin N, Ellidok H (2014). Çocuklarda Üst Çene Labial Frenulumun Klinik ve Diş Çürüğü Açısından Değerlendirilmesi. Turkiye Klinikleri Diş Hek Bilimleri Derg.

[ref14] Kirzioglu Z, Simsek S, Gurbuz T, Yagdıran A, Karatoprak O (2002). The prevalence of caries in 2-5 age group children and the evaluation of some risk factors in Erzurum, Bursa and Isparta provinces. J Fac Dent Ataturk Uni.

[ref15] Kotlow LA (2013). Diagnosing and understanding the maxillary lip-tie (superior labial, the maxillary labial frenum) as it relates to breastfeeding. J Hum Lact.

[ref16] Kotlow LA (2010). The influence of the maxillary frenum on the development and pattern of dental caries on anterior teeth in breastfeeding infants: prevention, diagnosis, and treatment. J Hum Lact.

[ref17] Nathan J (2017). The indications, timing, and surgical techniques for performing elective lingual and labial frenulectomies for infants and children. Inter J Otorhinolaryngology.

[ref18] Placek M, Skach M, Mrklas L (1974). Significance of the labial frenum attachment in periodontal disease in man. Part I. Classification and epidemiology of the labial frenum attachment. J Periodontol.

[ref19] Piovesan C, Mendes FM, Ferreira FV, Guedes RS, Ardenghi TM (2010). Socioeconomic inequalities in the distribution of dental caries in Brazillian preschool children. J Public Health Dent.

[ref20] Samuel SR, Acharya S, Rao JC (2020). School Interventions-based prevention of early‐childhood caries among 3–5‐year‐old children from very low socioeconomic status: two‐year randomized trial. J Public Health Dent.

[ref21] Taran PK, Özdemir Ş, Akarca B, Yüksel H (2023). Evaluation of the relationship between maxillary labial frenulum attachment types, periodontal health, and dental caries in preschool children. Evaluation.

[ref22] Tinanoff N, Palmer CA (2000). Dietary determinants of dental caries and dietary recommendations for preschool children. J Public Health Dent.

[ref23] Weatherwax JA, Bray KK, Williams KB, Gadbury‐Amyot CC (2015). Exploration of the relationship between parent/guardian sociodemographics, intention, and knowledge and the oral health status of their children/wards enrolled in a Central Florida Head Start Program. Int J Dent Hygiene.

